# Supplementary material for: Cancer-associated KBTBD4 mutations induce differentiation defects and confer a unique therapeutic vulnerability
Source: Cancer Gene Ther. 2026 Aug 10;33(9):1092–8. doi: 10.1038/s41417-026-01068-x (PMC13518216; doi:10.1038/s41417-026-01068-x)
Supplement: Supplementary file 1 [file 41417_2026_1068_MOESM1_ESM.docx]

**Supplementary materials**

# Cancer-associated KBTBD4 mutations induce differentiation defects and confer a unique therapeutic vulnerability

# Rohit Sivaprasad^1^, Kristijonas Žemaitis^1^, David Linfeldt^1^, Sudip Ghosh^2^, Anne de Snaijer^1^, Mattias Magnusson^1^, Fredrik Ek^3^, Jenny Hansson^2^, Agatheeswaran Subramaniam^1*^.

^1^Division of Molecular Medicine and Gene Therapy, Lund Stem Cell Center, Lund University, Lund, Sweden.

^2^Department of Experimental Medical Science, Lund Stem Cell Center, Lund University, Lund, Sweden.

^3^Chemical Biology & Therapeutics, Lund University, Lund, Sweden.

**Supplementary methods**

**Sample preparation for cellular proteome analysis**

HL60 cell lines transduced with over-expression constructs of either wildtype or P311PP mutant were collected in ice-cold phosphate buffer saline solution (PBS), centrifuged, and stored as dry pellets at -80 °C until further use. Pellets corresponding to 500,000 cells were solubilized in 50 mM ammonium bicarbonate (Sigma) with 0.1% RapiGest (Waters) to denature proteins and shaken on a thermomixer (Eppendorf) at 400 rpm for 15 minutes decreasing the temperature from 80°C to 56°C, followed by reduction of disulfide bonds with 0.1M dithiothreitol at 56°C, cysteine alkylation with 0.2 M iodoacetamide at room temperature in the dark, and digestion overnight at 37°C with sequencing grade modified trypsin (enzyme: protein ratio 1:50, Promega). Digested peptides were acidified with 10% trifluoroacetic acid (TFA) and RapiGest was precipitated by incubation at 37°C followed by centrifugation. Peptides were desalted with SepPak C18 columns (Waters), dried by vacuum centrifugation, and resuspended in MS loading buffer prior to LC-MS analysis.

**Liquid chromatography and mass spectrometry (LC-MS)**

LC-MS analyses were carried out on an Orbitrap Exploris 480 MS instrument with a reverse phase UltiMate 3000 UHPLC system via an EASY-Spray ion source equipped with FAIMS Pro (all Thermo Fisher Scientific). Peptide samples were analyzed by data-independent acquisition (DIA). Digested peptides were loaded onto a trap cartridge (Acclaim PepMap C18, 5 mm particle size, 0.3 mm inner diameter x 5 mm length, Thermo Fisher Scientific) and separated on an EASY-Spray analytical column (2 mm particle size, 75 mm inner diameter x 500 mm length, Thermo Fisher Scientific). Each sample was injected once and eluted with a linear gradient ranging from 2-19% Solvent B (0.1%FA in 80% ACN) over 80 min, 19-41% B over 40 min, 41-90% B over 5 min and held at 90-95% B for 5 min at a constant flow rate of 300 nl/min at 45°C. FAIMS compensation voltages (CV) were set to -45 and -60. The spray voltage was set at 2.1 kV and the ion transfer tube temperature was set at 275°C. For DIA analysis, peptides were analyzed with one full scan (350–1,400 m/z, R = 120,000) at a normalized AGC target of 300%, followed by 38 DIA MS/MS scans (350–1,050 m/z) in HCD mode (isolation window 22 m/z, 1 m/z window overlap, normalized collision energy 27%), with fragments detected in the Orbitrap (R = 15,000). All data were acquired in positive polarity and MS/MS were acquired in centroid mode.

**MS raw data processing and Statistical analysis**

The MS data were searched with ‘directDIA’ in Spectronaut (version 18, Biognosys AG) against the human SwissProt reference proteome and commonly used contaminants. Searches used carbamidomethylation as fixed modification, and methionine oxidation as well as protein N-terminal acetylation as variable modifications. The Trypsin/P proteolytic cleavage rule was used, permitting a maximum of 2 missed cleavages and a minimum peptide length of 7 amino acids. ‘Cross run normalization’ was enabled with Normalization Strategy set to ‘local normalization’ based on rows with ‘Identified in All Runs (Complete)’. Data filtering was set to Q-value and the Q‐value thresholds were set to 0.01 at PSM, peptide, and protein levels. Protein quantification and statistical analysis were performed with Msstats (1) (version 4.17.1). Contaminants were filtered and features were converted to MSStats format for downstream processing. Uninformative features were removed and missing values were imputed with the ‘MBimpute’ function within MSStats. Statistical analysis was performed with MSStats ‘group comparison’. Differentially expressed proteins were considered with adjusted p-value of less than 0.05 between groups for each comparison.

**Sequence**

The spacer sequence is provided below.

ACCGGTCCCGGCGTGACCGTGAAGGACGTGAACCAGCAGGAATTCGTCAGAGCCCTGGCCGCCTTTCTGAAGAAAAGCGGCAAACTCAAGGTGCCCGAGTGGGTGGACACCGTGAAGCTGGCCAAGCACAAAGAGCTGGCCCCCTACGACGAGAACTGGTTCTACACCAGAGCCGCCAGCACCGCCAGACACCTGTACCTGAGAGGCGGAGCCGGCGTGGGCAGCGTGACCAAGATCTACGGAGGAGTCGACGAATTCGGTACCGCGGGCCCGGGATC

**References**

1. Huang, T. et al. MSstatsTMT: Statistical Detection of Differentially Abundant Proteins in Experiments with Isobaric Labeling and Multiple Mixtures. Mol Cell Proteomics 19, 1706–1723 (2020).
2. Perez-Riverol Y, Bai J, Bandla C, Hewapathirana S, García-Seisdedos D, et al. The PRIDE database resources in 2022: A Hub for mass spectrometry-based proteomics evidences. Nucleic Acids Res. 2022;50:D543-D552.

**Supplementary figure legends**

**Supplementary Figure 1.** (A) Transduction efficiency of KBTBD4 mutants in HSC enriched CD34^+^CD38^-^CD90^+^CD45RA^-^ population and (B) the CD34^+^ bulk stem and progenitor cells. The bulk CD34^+^ cells were transduced with KBTBD4 mutants, and the frequency (C) and numbers (D) of progenitor (CD34^+^) and stem cells (CD34^+^ EPCR^+^) were measured at 7 days post transduction. One-way ANOVA was performed to assess significant differences and means between WT KBTBD4 and other groups were compared using post hoc test. (E) Colony forming capacity of stem and progenitor cells transduced KBTBD4 mutants. The HSPCs were transduced and expanded for 7 days and plated for 13 days to induce colony formation. Statistically significant difference was assessed using two-way ANOVA and post hoc tests were performed to compare means of each group in every sample to the corresponding group in WT KBTBD4 sample. Data are shown as the average of 3 replicates from 1 of 3 independent experiments with similar results. The p values are represented as * = ≤ 0.05, ** = ≤ 0.01, *** = ≤ 0.001, **** = ≤ 0.0001 in the figure and no asterisk is used for non-significant results (p value > 0.05).

**Supplementary Figure 2.** (A) A comparison of degradome profiles between the P311PP mutant and molecular glue UM171 as quantified in mass spectrometry (‘*’ denotes adjusted p-value < 0.05) (B) Viability of HL60 RCOR1-GFP cells with the co-treatment of UM171 and two class I specific HDAC inhibitors (mocetinostat and romidepsin) as well as three Pan HDAC inhibitors (belinostat, pracinostat and vorinostat). Cells were treated with the inhibitors for 3 days and the viability range was normalized to DMSO controls. Statistically significant differences were assessed using one-way ANOVA and the mean of all the groups were compared to the mean of UM171 using post hoc tests. Data from 3 replicates from 1 of 2 independent experiments with similar results are shown. In the figure, p value is represented as * = ≤ 0.05, ** = ≤ 0.01, *** = ≤ 0.001, **** = ≤ 0.0001 and no asterisk for non-significant results (p value > 0.05).

**Supplementary Figure 3.** (A) Viability of the modelled HSPCs treated with belinostat (125nM) and mocetinostat (250nM) for 5 days normalized to DMSO controls. (B) Erythroid (CD71^+^) and myeloid (CD11b^+^) lineage numbers in HSPCs expressing either spacer, wild type or KBTBD4 mutants and treated with either DMSO, belinostat (125nM) or mocetinostat (250nM) for 5 days. Statistical significance was tested using two-way ANOVA and post hoc tests were performed to compare mean of all groups to WT KBTBD4 treated with DMSO. (C) Lineage output in HSPCs treated with belinostat (125nM) and mocetinostat (250nM) for 5 days. Statistical differences were assessed using one-way ANOVA and mean of DMSO treated sample was compared to all other samples using post hoc tests. In the figure, p value is represented as * = ≤ 0.05, ** = ≤ 0.01, *** = ≤ 0.001, **** = ≤ 0.0001 and no asterisk for non-significant results (p value > 0.05). CD71-Erythroid marker; CD11b-Myeloid marker.

**Supplementary figures**

**Supplementary Fig 1**

**Supplementary Fig 2**

**Supplementary Fig 3**

**Supplementary table legends**

**Supplementary Table 1.** ELM2-GFP mean fluorescent intensity (MFI) values for RCOR1, RCOR3 and MIER3 ELM2 domains when co-expressed with individual KBTBD4 mutants. The columns, from left to right, show percentage of live cells that are GFP+, the percentage of live cells that are Kusabira orange+, MFI of GFP in the double positive populations (KuO+ GFP+), and the MFI normalized to the corresponding ELM2-GFP sample co-expressed with WT KBTBD4.

**Supplementary Table 2.** Quantitative proteomic analysis in HL60 cells transduced with P311PP mutant in comparison to the wild type KBTBD4.

**Supplementary Table 3.** Mutant ELM2 clones used in alanine scan. Amino acids in RCOR1 ELM2 domain were substituted with either alanine or glycine (where WT alanine was present).

**Supplementary tables**

**Supplementary Table 3**

| Position within RCOR1 ELM2 Domain | Wildtype Amino acid | Substituted Amino acid |
| --- | --- | --- |
| 6 | Y | A |
| 7 | Q | A |
| 8 | A | G |
| 9 | V | A |
| 10 | V | A |
| 11 | P | A |
| 12 | D | A |
| 13 | F | A |
